# Mental well-being and patient activation during the first eight months of the COVID-19 pandemic in Denmark - a cohort study among 710 Danish adults with chronic conditions

**DOI:** 10.1186/s12889-023-16316-0

**Published:** 2023-08-02

**Authors:** Berit Kjærside Nielsen, Pernille Bjørnholt Nielsen, Caroline Trillingsgaard Mejdahl, Lise Arnth Nielsen, Camilla Palmhøj Nielsen, Helle Terkildsen Maindal, Michael Wolf

**Affiliations:** 1 DEFACTUM – Public Health Research, Central Denmark Region, Olof Palmes Allé 15, Aarhus N, DK-8200 Denmark; 2 Department of Public Health - Department of Health Services Research, Aarhus University, Aarhus C, Denmark; 3 Institute for Public Health and Medicine (IPHAM) - Center for Applied Health Research on Aging, Northwestern University Feinberg School of Medicine, Chicago, IL USA

**Keywords:** COVID-19, Well-being, Patient activation, Chronic conditions, Questionnaire study

## Abstract

**Background:**

COVID-19 has highlighted the importance of patient activation in managing chronic conditions and promoting resilience during times of crisis. Patient activation refers to an individual’s knowledge, skills, and confidence in managing their own health and healthcare. Previous research has shown that people with higher levels of patient activation are better prepared to navigate the challenges of chronic illness and are more likely to engage in healthy behaviors. However, the impact of patient activation on COVID-19-related concerns and mental well-being among people with chronic conditions during the pandemic remains unclear. This study aims to investigate the possible role of patient activation in shaping COVID-19-related concerns and to describe changes in mental well-being among Danish adults with one or more chronic conditions during the early months of the pandemic.

**Methods:**

Danish adults with chronic conditions (e.g. diabetes, coronary heart disease, obstructive pulmonary lung disease, cancer) who had participated in a municipal health education program prior to the COVID-19 outbreak were asked to participate in this prospective questionnaire study in May 2020 and November 2020. Sociodemographic (sex, age, living status, educational attainment, employment status) and disease-related information (diagnosis, one or more chronic conditions) along with the Patient Activation Measure were collected before the outbreak and were obtained from a clinical database used for monitoring and evaluation of municipal health education programs. In contrast, the two questionnaires collected six months apart consisted of single items related to concerns about COVID-19 and the WHO-5 well-being index.

**Results:**

A total of 710 people with chronic conditions (mean age 60.9 years; 55.8% female) participated at both time points. In bivariate analyses, patient activation was associated with COVID-19-related concern and well-being. At follow-up, participants experienced a significant decrease in well-being. The decrease was associated with poorer well-being measured six months earlier, a greater perception that it had become more challenging to take care of one’s health due to the pandemic, and finally, feeling lonely. The association between patient activation and well-being ceased to be significant in the multivariate regression model.

**Conclusions:**

A considerable proportion of people with chronic conditions participating in this study have been mentally burdened during COVID-19. Although lower levels of patient activation were associated with greater COVID-19-related concerns, it did not have a significant impact on mental well-being over time.

## Introduction

The severe and acute coronavirus (SARS-CoV-2) resulting in the COVID-19-disease (COVID-19) spread during the winter of 2019–2020 and was declared a global pandemic as of March 2020 [1]. Since the discovery of the virus, more than three million cases have been reported in Denmark, while over 8.000 have died from COVID-19 [2]. Denmark experienced the first lockdown in March 2020. In September 2020, a second wave of COVID-19 infections hit and a following lockdown began in December 2020. From early on, the pandemic required people worldwide to listen to and understand rapidly changing information on public health and to be able to take swift action to minimize their likelihood of contracting or transmitting the virus. The Danish government implemented various measures to slow down the spread of the virus and introduced several unprecedented restrictions with a significant impact on people’s everyday life, including restrictions on public gatherings. In addition, non-essential health services such as health education programs and rehabilitation, were paused indefinitely [3]. The introduction of physical distancing at the beginning of the pandemic was a crucial precaution to reduce the spread of COVID-19. However, as governments attempted to reduce the outbreak by implementing containment measures, health researchers and clinical experts started to worry about the indirect health impacts beyond those caused by the virus per se. Thus, mental health was given increased focus, and the greater part of the studies find that the pandemic impacted people’s mental health in a negative way. Furthermore, people with pre-existing chronic conditions or those at a higher risk of severe illness has been found to be more susceptible to adverse effects on their mental well-being as a result of the COVID-19 pandemic. This includes depression, anxiety, and sleep problems [4–9], loneliness [10–12], and fear and worry related to the pandemic [13, 14].

Studies undertaken during the pandemic has also revealed that the alterations brought about by government restrictions had a significant impact on peoples’ engagement in daily health behaviors [15]. People with chronic conditions who may experience challenges with managing their physical and mental health are more vulnerable than others to a potential worsening of their condition because of disruptions to care [16]. Having the knowledge, skills, and confidence to manage disease and health problems can collectively be referred to as patient activation, and studies suggest that people move through different levels from low (disengaged and overwhelmed) to high (maintaining new behaviours) activation [17]. Those with chronic conditions with higher levels of activation are more likely to engage in healthy behaviours that promote self-management and prevention of further illness [18, 19]. Moreover, patient activation is a reliable psychosocial determinant of mental and physical health [20, 21]. A longitudinal study found that lower levels of patient activation were associated with symptoms of depression and lower health-related quality of life [22].

Only a few studies have examined the role of patient activation in light of COVID-19 [23–26]. Therefore, the aim of the current study was twofold: (1) to investigate the possible role of patient activation on COVID-19-related concerns, and (2) to describe changes in mental well-being among Danish adults with one or more chronic conditions during the early months of the COVID-19 pandemic.

## Methods

### Study design and participants

The PAM-COVID-19 survey is a prospective cohort study of people living with chronic conditions during COVID-19. Data on eligible participants were extracted from MoEva 2.0, a monitoring and evaluation database used to collect standardized and comparable patient-reported outcome data from health education programs in municipalities in Denmark [27]. Participants from ten municipal health services were screened for eligibility on three criteria’s: (1) Adults > 18 years of age who within the last 12 months (March 2019 - March 2020) had participated in a municipal health education program, (2) had one or more chronic conditions, and (3) had completed the Patient Activation Measure (PAM) within the past 12 months and prior to the COVID-19 outbreak (hereby defined as pre-COVID-19 measure). After an initial assessment, the citizen is offered to attend a health education program of eight to twelve weeks in duration, which may include courses on diabetes, cardiovascular disease, chronic obstructive pulmonary disease, obesity, stress, or mental health. When identified, eligible participants were sent an invitation letter, a written consent form, and the PAM-COVID-19 survey in their e-Boks, a trusted Nordic provider of secure platforms and digital postboxes. Two reminders were mailed, and subsequently, a paper version was sent to the address of non-responders to increase participation from eligible participants who could not receive the mail digitally (e.g. do not have access to a computer, low digital literacy). A total of 2.147 adults were found eligible, and the survey was sent out on May 4th 2020 (survey 1), after seven weeks of lockdown. To assess changes in mental well-being and COVID-19-related concerns over time, a second questionnaire was sent out to participants on November 18th 2020, six months after the initial survey, during the ongoing COVID-19 pandemic (survey 2).

### Measurements

Pre-COVID-19 measures were extracted from the MoEva 2.0 database for eligible participants and.

consisted of sociodemographic and disease variables: sex (female/male), age (continuous), living status (living alone/living with partner or children), educational attainment (low: 0–10 years of education/medium: 11–14 years of education/high: ≥15 years of education), employment status (employed/unemployed), and multimorbidity (one chronic condition/> 1 chronic condition). Due to few cases in the low group, educational attainment was dichotomized in the analyses (low/medium vs. high). Patient activation was measured prior to COVID-19 using the 13-item Patient Activation Measure (PAM). PAM is a validated tool that measures a person’s knowledge, skill, and confidence to engage in the process of one’s care and self-management [17, 28]. PAM is a valid and reliable measure that is associated with outcomes across many chronic conditions, and it has been translated and validated in a Danish setting [29–34]PAM is scored on a scale from 0 to 100, with a greater value indicating a higher level of activation, and can be categorized into four activation levels. Level 1 (0.0–47.0): disengaged and overwhelmed; Level 2 (47.1–55.1): becoming aware but still struggling; Level 3 (55.2–72.4): taking action; and Level 4 (72.5–100): maintaining behaviours, but may not necessarily sustain them under stress. When Denmark implemented a lockdown on March 11, 2020, a total of 275 eligible participants (38.7%) had completed a health education program and had a PAM filled out after the completion of the program. Meanwhile, 435 eligible participants (61.3%) were either in the process of undertaking a health education program or had not yet started one, and thus possessed a PAM from before the commencement of the program.

The primary outcome was mental well-being measured eight months into the pandemic in Denmark (survey 2). The WHO-5 is a five-item self-report measure of mental well-being. It consists of five questions indicating the extent to which respondents have been feeling well mentally during the last two weeks. Each question is scored on a five-point Likert scale indicating how often respondents have experienced specific feelings. The points are added and multiplied by four, calculating the total score ranging from 0 to 100; higher scores indicating higher level of well-being. A score below 50 is considered reduced mental well-being and risk of depression. The WHO-5 well-being scale is believed to be a strong patient-related measure of mental well-being for chronic conditions [35, 36].

No standard questionnaire had existed for measuring COVID-19-related concerns at the beginning of the pandemic. Therefore, single items were developed specifically for the purpose of this study or adapted from ongoing national and international studies [23, 37]. These included questions about whether they believed their everyday actions had a saying in getting COVID-19 as well as COVID-19 related worry, containing feeling stressed, having trouble sleeping, and feeling lonely. Table 2 lists the COVID-19 single items when they were measured and the response format. Finally, participants were invited to write additional comments in an unlimited text box, subsequently used for thematic analysis. Results have been reported elsewhere [38].

### Statistical analysis

Descriptive statistics were calculated to portray the sociodemographic and disease status of the participants. Mean scores were calculated for the PAM, and associations with COVID-19-related concerns were examined using an independent-samples t-test, one-way analysis of variance and Pearson’s correlations. To explore potential differences in demographic characteristics and PAM levels between responders and non-responders, we conducted non-response and drop-out analyses using independent samples t-test and chi-square (χ^2^) tests. Responders were defined as participants who completed both surveys, while non-responders were defined as those who did not complete the first survey. Dropouts were defined as participants who completed the first survey but did not respond to the follow-up survey. A paired samples t-test was conducted to detect changes in the WHO-5 well-being index from survey 1 to survey 2. Next, we conducted unadjusted and multivariate linear regression analyses to examine associations between various sociodemographic variables, disease variables, COVID-19-related concerns, and well-being at follow-up (survey 2). In the analyses, we used the responses to COVID-19-related items as they were reported in survey 2. Only variables significantly associated with the dependent variable in bivariate analyses (< 0.05) were entered into the multivariate analysis while controlling for the potential confounding effects of age, sex, and educational attainment. When examining the relationship between variables, correlation coefficients were used to measure the strength and direction of the linear relationship. Commonly used thresholds for interpreting the strength of correlations are as follows: negligible when r_s_ < 0.3, low when 0.3 ≤ r_s_ < 0.5, moderate when 0.5 ≤ r_s_ < 0.7, high when 0.7 ≤ r_s_ < 0.9, and very high when 0.9 ≤ r_s_ ≤ 1 [39]. Given the relatively large sample size and few missing items on scale variables, list-wise deletion was chosen [40]. Statistical significance was considered when p-values were < 0.05 (two-sided). Statistical analyses were conducted using IBM SPSS Statistics v20.

### Ethics

There is no formal agency for ethical approval of questionnaire-based studies in Denmark. 

Before conducting the study, we obtained approval from the Danish Patient Safety Authority that personally identifiable data could be retrieved from the MoEva 2.0 database. The study was registered at the Central Denmark Region’s research notification system (journal number: 1-16-02-179-20). Participants in the study gave electronic or written consent and were informed that participation was voluntary and that they could withdraw from the study at any time. Participants did not receive any financial compensation for their partaking.

## Results

### Sample

**Fig. 1:**
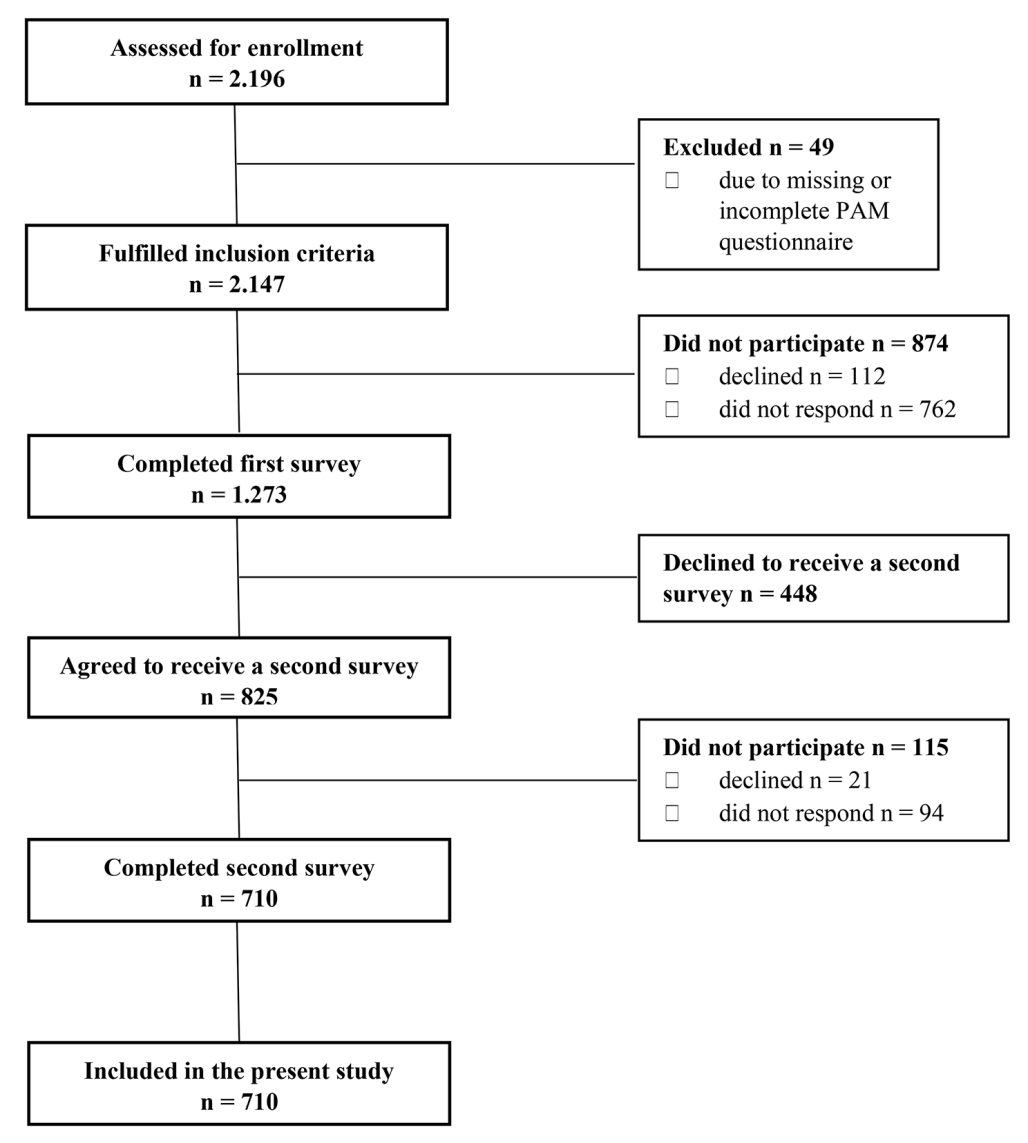
Flowchart of the PAM COVID-19 cohort sample

A total of 1273 (59%) out of 2147 eligible participants completed survey 1 [41]. Of those who entered the study at baseline, 710 people (55.8%) completed survey 2 and were included in the present study. See Fig. 1. The mean age of the participants was 60.9 years (SD = 11.4, range = 19–89 years), 55.8% were female, the majority (73.8%) lived with another person, nearly half (48.7%) had medium educational attainment, and about one-third (37.9%) indicated that they were in paid employment. The most prevalent chronic conditions were Type 2-diabetes (19.9%), cardiovascular disease (18%), chronic obstructive pulmonary disease (14.9%), overweight (13.7%), and cancer (13.4%), and almost half of the participants (43.7%) were living with two or more chronic conditions. See Table 1. The mean PAM score was 60.9 (SD = 13.5), and 16.9% were categorized as PAM level 1, 20.8% as level 2, 39% as level 3, and 23.2% as level 4. Compared to those who only participated in survey 1 (n = 563), those participating in both surveys were more likely to be younger, have higher patient activation, and higher educational attainment (p < .05). No statistically significant differences were found regarding sex, multimorbidity, and well-being.

**Table 1:** Participant characteristics (n = 710)

Variables	n (%)
Age (Mean (SD))	60.9 (11.4)
Sex	
Female	396 (55.8)
Male	314 (44.2)
Living situation	
Living alone	152 (21.4)
Living with partner/children	520 (73.2)
Not specified	38 (5.4)
Educational level	
Low	87 (13.5)
Medium	346 (53.7)
High	211 (32.8)
Not specified	66 (9.3)
Employment status	
Paid employment	269 (39.0)
Not working (retired/unemployed)	420 (61.0)
Not specified	21 (3.0)
Single or multiple chronic conditions	
One chronic condition	355 (53.0)
More than one chronic condition (multi disease)	310 (47.0)
Not specified	45 (6.3)
Chronic conditions	
Cancer	95 (13.5)
Cardiovascular disease	128 (18.1)
Chronic obstructive pulmonary disease	106 (15.0)
Chronic pain	16 (2.3)
Diabetes (type 2)	144 (20.3)
Mental health (anxiety/depression)	9 (1.3)
Musculoskeletal disorder	11 (1.6)
Osteoporosis	9 (1.3)
Overweight	97 (13.7)
Stress	45 (6.4)
Other conditions	46 (6.5)
Patient Activation Measure (PAM)	
Patient activation score (Mean (SD))	60.9 (13.5)
- Level 1	120 (16.9)
- Level 2	148 (20.8)
- Level 3	277 (39.0)
- Level 4	165 (23.2)

### Patient activation and COVID-19-related concern

Table 2 summarizes the participants’ responses to COVID-19-related concerns at both time points. Participants who had their health education programme interrupted due to the COVID-19 outbreak (M = 59.4, SD = 12.3) compared to those who had completed their health education programme (M = 63.4, SD = 15.1) had significantly lower PAM scores, t (708) = -3.7, p = .000. Furthermore, participants who lived alone (M = 58.9, SD = 12.2) compared to those living with a partner and/or children (M = 61.8, SD = 12.8) had significantly lower PAM scores, t (684) = -2.5, p = .013. There was also a statistically significant effect of educational attainment on PAM, showing that participants with high educational attainment (M = 63.8, SD = 13.4) had higher PAM score than participants with low or medium educational attainment (M = 59.9, SD = 13.5), t (642) = -3.4, p = .001. Finally, participants in paid employment (M = 63.3, SD = 12.8) compared to participants who were unemployed (M = 59.7, SD = 13.9) had statistically significantly higher PAM scores, t (687) = 3.4, p = .001. Other participant characteristics such as sex, age, and having one or more chronic conditions were not associated with the level of PAM (p > .05).

**Table 2:** COVID-19 concern

			n (%)	Mean (SD)	*P* value	*PAM correlation*	*P* value	
**Measured in the first survey**	**Survey**							
I believe to be at particular risk of getting seriously ill if I get the coronavirus	Survey 1	Yes	401 (56.7)					
		No	131 (18.5)					
		I don’t know	175 (24.8)					
**Measured in both surveys**								
I was tested positive with the coronavirus	Survey 1	Yes	44 (3.6)					
	Survey 2	Yes	16 (2.3)		0.336			
My actions will influence whether or not I get the coronavirus	Survey 1	10-point Likert scale*	674	8.34 (2.2)		0.12	**0.002**	
	Survey 2		700	8.58 (1.9)	0.113	0.06	0.089	
It has become more difficult to take care of my own health due to the coronavirus	Survey 1	10-point Likert scale*	653	5.04 (2.9)		-0.05	0.196	
	Survey 2		653	5.35 (2.9)	**0.015**	-0.1	**0.002**	
Over the past two weeks, how often have you felt nervous or “stressed” because of the coronavirus?	Survey 1	6-point Likert scale**	710	4.78 (1,2)		0.12	**0.002**	
	Survey 2		702	4.86 (1.2)	0.146	0.11	**0.003**	
Over the past two weeks, how often have you had trouble falling asleep, because you were thinking about the coronavirus	Survey 1	6-point Likert scale**	709	5.49 (1.0)		0.12	**0.002**	
	Survey 2		702	5.54 (0.9)	0.116	0.09	**0.018**	
Over the past two weeks, how often have you felt alone or lonely because of the coronavirus?	Survey 1	6-point Likert scale**	710	4.91 (1.4)		0.22	**0.000**	
	Survey 2		701	5.08 (1.2)	**0.000**	0.20	**0.000**	

* 10-point Likert scale: 1 = Strongly disagree – 10 = Strongly agree.

** 6-point Likert scale: 1 = All the time – 6 = Never.

Patient activation measured prior to the COVID-19 outbreak was associated with participants’ perceived risk of severe illness from COVID-19 *F*(2, 704) = 6.71. Specifically, participants who perceived themselves to be at high risk of severe illness had significantly lower PAM scores compared to those who did not perceive themselves to be at high risk (p = .000). Participants (n = 175) who answered that they were in doubt as to whether they were at risk did not differ from those who believed themselves to be at high risk and those who did not (p > .05). Although small, correlations between PAM and COVID-19-related concern were statistically significant in all variables, r(670–710) = 0.10-0.21, p < .05. However, believing that one’s actions influence whether or not you would contract the virus was only statistically significant associated to higher PAM score in survey 1 and ceased to be significant in survey 2. This pattern was the opposite of whether it had become more challenging to care for one’s health due to the coronavirus. Only when asked in survey 2, the association between a higher PAM score and the perception that it had become more challenging to take care of one’s health due to the coronavirus was statistically significant (p < .05).

### Development in well-being

Approximately 22% (159/704) of the sample reported values below 50 on the WHO-5 well-being index in survey 1, indicating a potential risk of depression. This number had increased to 28% (197/702) in survey 2. Looking at the mean values among participants with different levels of patient activation, we found that those with the lowest levels of patient activation had lower well-being (PAM level 1: mean WHO-5 score 54.8 (survey 1), 52.2 (survey 2); PAM level 2: mean WHO-5 score 63.4 (survey 1), 59.5 (survey 2)) compared to those with higher levels of patient activation (PAM level 3: mean WHO-5 score 67.2 (survey 1), 63.6 (survey 2); PAM level 4: 73.2 (survey 1), 70.1 (survey 2)). A paired-samples t-test was conducted to analyze changes in well-being from survey 1 to survey 2. For the whole sample, a small but significant decrease in well-being was seen from survey 1 (M = 65.76, SD = 20.95) compared to survey 2 (M = 62.37, SD = 22.11); t (695) = 4.90, p = .000). However, the effect size was very small (d = 0.16).

### Predictors of change in well-being

Changes in well-being are presented in Table 3. The linear regression model was significant to predict well-being at follow-up (survey 2); F(11, 600) = 48.64, p < .000. R^2^ for the overall model was 47%, with an adjusted R^2^ of 46%. The model revealed that a decrease in well-being at follow-up was associated with poorer well-being measured six months earlier (survey 1), less belief that your actions could influence whether or not you would contract COVID-19, a stronger feeling that it had become more difficult to take care of one’s health due to the pandemic, and finally, feeling more alone or lonely because of the pandemic. None of the sociodemographic variables was associated with a change in well-being, nor were the variables feeling stressed or having trouble falling asleep. Finally, in the multivariate regression model, although a tendency was seen, the association between PAM and changes in well-being was no longer statistically significant (p = .06).

**Table 3:** Unadjusted and adjusted linear regression models examining predictors of change in well-being during the COVID-19 pandemic*

Explanatory variable	Unadjusted analysis			Adjusted analysis		
	B	95% CI	*p*-value	B	95% CI	*p*-value
Sex (0 = male, 1 = female)	-0.101	-7.771 – -1.193	**0.008**	-0.011	-3.230–2.230	0.719
Age	0.159	0.167–0.453	**0.000**	0.021	-0.083–0.166	0.514
Living status (alone = 0, live with partner/children = 1)	0.095	1.042–8.968	**0.013**	-0.022	-4.370–2.077	0.485
Educational attainment (low/medium = 0, high = 1)	0.102	1.148–8.450	**0.010**	0.017	-1.967–3.572	0.570
Work status (employed = 0,unemployed = 1)	-0.054	-5.849–0.973	0.161	-	-	-
Chronic condition (one = 0, > 1 = 1)	-0.074	-6.688–0.096	0.057	-	-	-
WHO-5 (survey 1)	0.640	0.615–0.736	**0.000**	0.484	0.434–0.580	**0.000**
Patient Activation Measure	0.248	0.287–0.522	**0.000**	0.059	-0.005–0.195	0.062
My actions will influence whether or not I get the coronavirus	0.163	1.053–2.786	**0.000**	0.104	0.507–1.915	**0.001**
It has become more difficult to take care of my own health due to the coronavirus	-0.269	-2.646 – -1.543	**0.000**	-0.094	-1.223 - -0.248	**0.003**
Over the past two weeks, how often have you felt nervous or “stressed” because of the coronavirus?	0.362	5.570–8.200	**0.000**	0.067	-0.196–2.741	0.089
Over the past two weeks, how often have you had trouble falling asleep, because you were thinking about the coronavirus	0.310	5.974–9.492	**0.000**	0.034	-1.030–2.748	0.372
Over the past two weeks, how often have you felt alone or lonely because of the coronavirus?	0.427	6.428–8.827	**0.000**	0.151	1.343–3.998	**0.000**

*Statistically significant values are shown in **bold**

## Discussion

In this prospective study, we used data on patient activation collected before the pandemic outbreak to investigate the possible effect of patient activation on COVID-19-related concerns and mental well-being. Our findings showed a weak correlation between patient activation and participants’ level of COVID-19-related concern. Specifically, those who reported feeling less stressed, experiencing no sleep disturbance, and feeling less lonely had lower levels of patient activation. The rather weak correlation between patient activation and COVID-19-related concerns observed in our study could be explained by societal changes related to the pandemic that may have affected all individuals regardless of their level of patient activation. For example, measures such as physical distancing and lockdowns may have limited opportunities for social interaction, leading to increased feelings of loneliness and stress among all participants. These external factors may have overshadowed the influence of patient activation on COVID-19-related concerns. The finding that people with higher patient activation were more likely to believe that their actions could influence their risk of infection is consistent with the broader literature on health beliefs, particularly the construct of self-efficacy that refers to an individual’s belief in their ability to successfully complete a specific task or achieve a desired outcome [42]. In the context of patient activation, self-efficacy is likely to play an important role in shaping peoples’ attitudes and behaviors related to health. People who are confident in their ability to take action to improve their health are likely to be more engaged and proactive in managing their health. Several studies have investigated the relationship between COVID-19 infection and access to and comprehension of health information, including factors such as knowledge, attitudes, and concern [11, 23, 43, 44]. However, apart from the present study, only a few studies have investigated patient activation in light of COVID-19 [23–26]. A study among people with Parkinson’s disease found that patients’ activation levels were inversely correlated with increased assistance for activities of daily living, increased tiredness, worsening symptoms, and lack of support from family and friends [25]. Another study by Imeri et al. found that participants who reported facing difficulties in managing their chronic conditions because of worry or fear over COVID-19 had lower levels of patient activation than those not affected by COVID-19-related worry or fear [24]. Finally, a study by Bronheim et al. found that high patient activation may ease the disruptive effects of external stress caused by the COVID-19 pandemic on patients’ ability to perform usual activities [26].

In the present study’s overall sample, a statistically significant decrease in well-being was observed six months after the first assessment. However, the small effect size indicated no clinically relevant worsening. When surveyed in the early weeks of the pandemic, more than one-fifth of the participants reported low levels of well-being, indicating risk of depression. This finding may represent a normal response to an unforeseen, traumatic event, which was then followed by a period of psychological adaptation and resilience [45]. A study investigating loneliness and mental health among older adults in Holland during the pandemic shows that mental health remained roughly stable [46]. Similar results are presented by Röhr et al., who found that the mental well-being of the German older population was largely unaltered during the COVID-19 lockdown [47]. Mergel et al. found that participants with a chronic mental disorder showed high stability over time and no other detrimental effects on mental health four weeks after lockdown, indicating a high resilience to the official restrictions and the pandemic itself [48]. While adaptation or status quo in mental well-being seemed to be the average reaction in the overall sample of the present study, there were smaller groups at risk of experiencing a more prolonged negative impact on mental well-being due to the COVID-19 pandemic. Lower well-being reported six months prior, less belief that your actions would influence whether or not you contracted COVID-19, a stronger feeling that it had become more challenging to take care of one’s health due to the pandemic, and finally, feeling lonelier because of the pandemic was associated with the strongest decrease in well-being at follow-up. Several studies have undeniably documented that the pandemic has impacted people’s mental health in a negative way, including those with a high risk of getting seriously ill if they contract COVID-19. A study conducted in the UK found that nearly half of the sample of people at high risk of severe illness due to COVID-19 reported a worsening of their mental health during the COVID-19 lockdown [49]. The same conclusion is drawn in a Swedish study, which also finds that up to half of the participants had decreased mental health in terms of feeling depressed and having sleep disturbances [4]. Our study showed that the association between patient activation and well-being ceased to be significant in the multivariate analyses. However, in the non-responder analyses, we found that those who participated in both surveys were more likely to have higher patient activation than those who only completed the first survey, which may have impacted the result. Generally, studies tend to show an association between higher patient activation and increased well-being [22, 50, 51]. Although we did not find a statistically significant effect of patient activation on mental well-being in the final model, a tendency was seen, and it may be related to the ability of those with high patient activation being able to pull through the negative impact of the pandemic.

The strengths of this study included the large sample size. Secondly, the longitudinal design made it possible to assess the association between patient activation and COVID-19-related worry over time and to examine factors associated with a change in mental well-being. This study, however, also has its limitations. Participants in our study, who had only just started or were not yet enrolled in a health education program at the onset of the pandemic in general had lower levels of patient activation compared to those who had completed a program. However, participation in a health education program was not part of the narrative in the study as we solely focused on the potential impact of PAM levels on COVID-19 management. Differences in responders and non-responders in the first survey and those who participated in both surveys compared to those who dropped out after the first survey could also present a potential bias. On the other hand, we were able to identify eligible participants from the MoEva 2.0 database consisting of individual-level register data on sociodemographic information and their pre-COVID patient activation score. Therefore, we could check for and report potential non-response bias, which can occur when people who refuse to take part in a study are systematically different from those who participate. Many studies initiated at the beginning of the COVID-19 pandemic consisted of online surveys using non-probability and convenience samples. In order to understand prevalence in a population, it is important to know who the respondents and the non-respondents are [52].

## Conclusion

In conclusion, this study showed that a considerable proportion of individuals with one or more chronic conditions experienced mental burden during the COVID-19 pandemic. Notably, the negative impact on well-being was most pronounced among individuals who initially had lower well-being, perceived greater challenges in managing their health due to the pandemic, and felt increased loneliness as a result of it. Additionally, the study suggests a potential link between lower levels of patient activation and more pronounced negative effects.

### Implications

Knowledge of the significance of lockdowns during the COVID-19 pandemic on different groups of people is crucial if we are to learn what hinders compared to what increases the risks of prolonged reduced well-being. This is specifically the case for people with chronic conditions, given disease-related issues that need regular and ongoing healthcare support. Future research should further explore the complex interplay between patient activation and external factors in shaping individuals’ responses to health crises such as the COVID-19 pandemic.

## Data Availability

The datasets generated during the current study have been obtained under specific agreements that impose restrictions on its distribution or public availability but are available from the corresponding author on reasonable request.
